# Uterine Leiomyosarcoma in a 22-Year-Old Young Woman: A Case Report

**DOI:** 10.7759/cureus.62087

**Published:** 2024-06-10

**Authors:** Mohammed Bendimya, Fatima Rezzoug, Mouhsine Omari, Ouissam Al Jarroudi, Sami Aziz Brahmi, Said Afqir

**Affiliations:** 1 Medical Oncology, Mohammed VI University Hospital, Faculty of Medicine and Pharmacy of Oujda, Mohammed First University, Oujda, MAR

**Keywords:** adjuvant chemotherapy, surgery, immunohistochemistry, uterine sarcoma, leiomyosarcoma

## Abstract

Leiomyosarcoma is one of the rarest types of gynecological cancer. It is a relatively rare condition that affects young women. The most frequent symptom of this disease is vaginal bleeding. The primary treatment for localized disease is still surgical intervention. It is widely recognized that leiomyosarcoma has a poor prognosis, with reduced survival rates and a high likelihood of early recurrence. This report presents a case of uterine leiomyosarcoma in a 22-year-old female patient. Following a total hysterectomy and bilateral salpingo-oophorectomy, the diagnosis of leiomyosarcoma was confirmed through a histopathological examination of the surgical specimen.

## Introduction

Leiomyosarcoma, a rare and malignant tumor derived from the uterine smooth muscle, comprises only 1% of all uterine malignancies [1]. These tumors are characterized by their aggressive behavior and are associated with a dismal prognosis, a high incidence of local recurrence, and metastasis [2]. Leiomyosarcoma primarily affects perimenopausal women, although it is rarely reported in younger women [3]. Some patients may exhibit symptoms indicative of large pelvic masses, such as hemorrhage or pressure sensations in the vaginal or abdominal areas. However, it's important to note that some individuals may remain entirely asymptomatic [1,2]. The diagnosis of leiomyosarcoma before surgery is challenging due to its resemblance to benign uterine leiomyomas [4]. The rarity of this condition limits our understanding of prognostic factors and optimal adjuvant treatments [5]. Therefore, the stage of the disease is considered the most critical determinant of the prognosis.

## Case presentation

**Figure 1:**
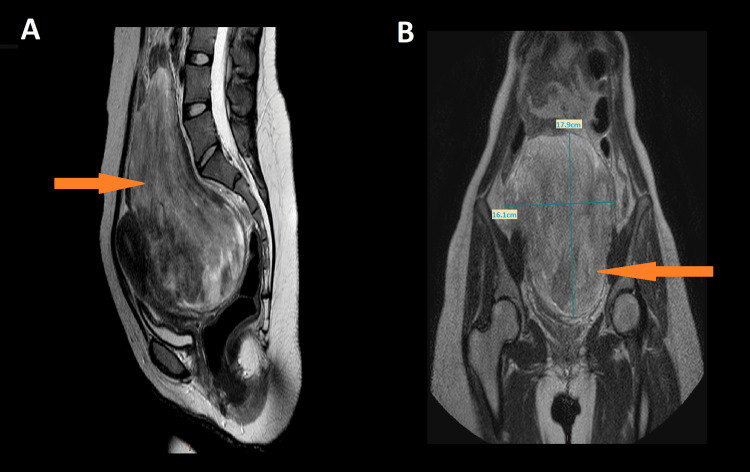
Magnetic resonance imaging of a young woman with uterine leiomyosarcoma (arrow) Sagital T2(A) and Coronal T2(B) show a large uterine mass measuring 18 x 16 x 11.5 cm, with hypointense and hyperintense signals on T2.

**Figure 2:**
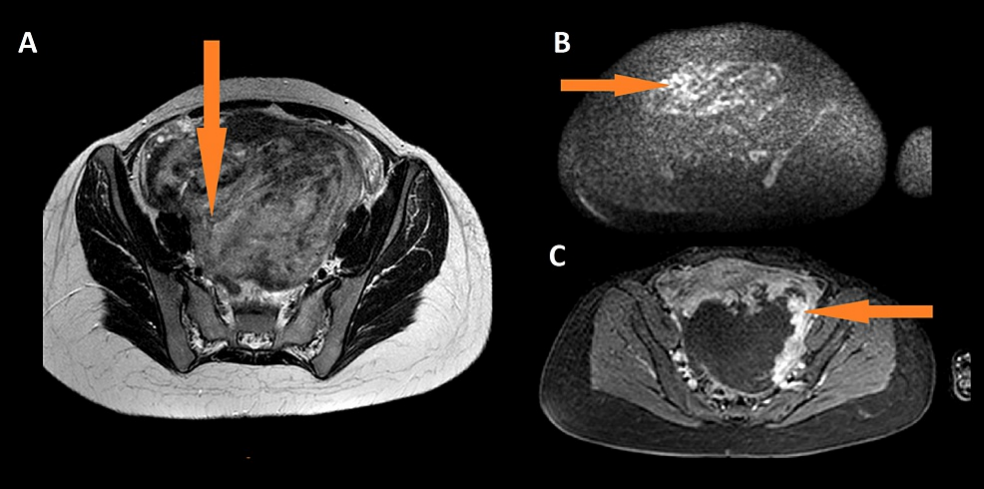
Axial T2(A), axial diffusion(B), and axial T2 C+(C) magnetic resonance imaging (arrow) Showing heterogeneous signal on T2 with hyperintense signal on diffusion, and intense peripheral enhancement.

In this case report, we describe a 22-year-old nulliparous woman with psychomotor retardation and no other concomitant malformations who presented with a rapidly growing abdominal mass and lower abdominal pain over a period of two months, along with lower abdominal pain. She had an excellent performance status (PS) of 0, and her prior periods had flowed normally and regularly. On the physical examination, there was a palpable mass arising from the pelvis to the epigastrium. Ultrasonography revealed a lateral uterine mass measuring 18 cm. Magnetic resonance imaging (MRI) of the pelvis was performed and described a voluminous posterior isthmus uterine mass measuring 18 x 16 x 11.5 cm, with hypointense and hyperintense signals on T2, hyperintense on diffusion, with a low apparent diffusion coefficient (ADC) of 0.5, and intense peripheral enhancement with a type 3 dynamic curve (Figures 1, 2).

**Figure 3:**
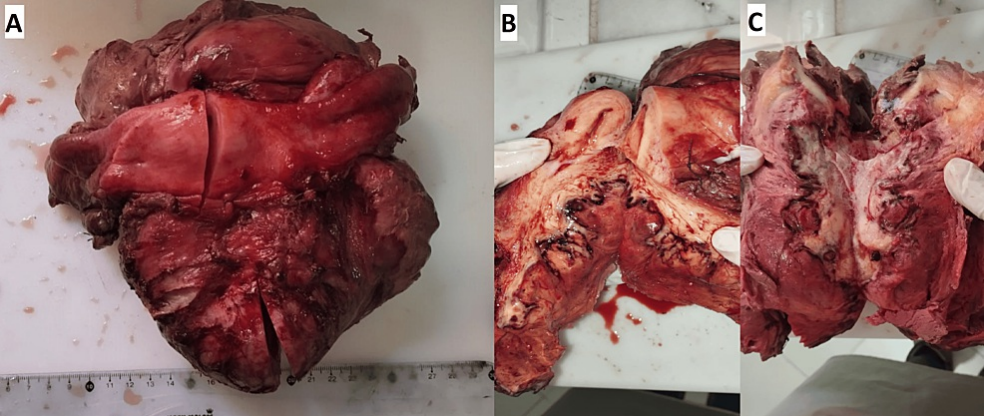
Macroscopic images of the resected mass A: Macroscopic image of a total hysterectomy with bilateral annexectomy; B, C: A cut section showed a large, well-circumscribed tumor with hemorrhage and necrotic areas.

This case was deliberated upon during our multidisciplinary tumor board (MDT) gathering, and the board's consensus choice was to carry out a total hysterectomy and bilateral salpingo-oophorectomy (Figure 3A).

**Figure 4:**
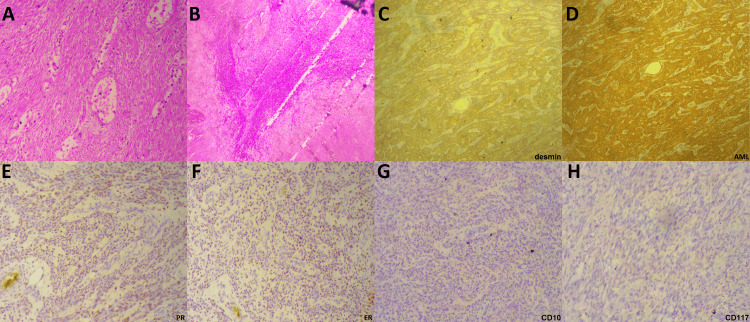
Microscopic and immunohistochemical findings of uterine leiomyosarcoma A, B: Microscopic examination showing fascicles of eosinophilic spindled cells with blunt-ended nuclei reveals variable pleomorphism with abundant eosinophilic cytoplasm and elongated, heterogeneous chromatin, with the nucleolus showing moderate atypia and some figures of mitosis with more than 10 mitoses per ten fields with focal necrosis (HE); C, D: Intense and diffuse positive staining of tumor cells by anti-desmine and AML antibodies (immunohistochemical staining, x200); E, F: Intense positive immunostaining of tumoral cells for PR and focal for ER (200×); G, H: Images showing the lack of staining of tumor cells by the anti-CD10 and anti-CD117 antibodies (immunohistochemical staining, x400).

The pathological examination revealed a massive tumor, measuring 21 cm in its largest dimension, which was removed in one piece (Figures 3B, 3C), infiltrating the uterine body and giving it a sarcomatous appearance. Microscopically, the tumor section displayed fusocellular tumor proliferation, featuring minimal to moderate atypia, occasional mitotic figures greater than 10 mitoses per 10 high-power fields (HPF), and several tumor necrosis areas. Perineural neoplastic invasion or vascular emboli were absent, with free resection margins. The immunohistochemical study exhibited intense and diffuse positive staining for AML, desmin, and both estrogen and progesterone receptors, with negative staining for CD10, CD117, C-Kit, and S100 (Figure 4).

The final diagnosis was uterine leiomyosarcoma. The patient's postoperative course was favorable, and she promptly commenced adjuvant chemotherapy with a combined regimen of gemcitabine 900 mg/m2 (IV) on day one and eight and docetaxel 100 mg/m2 (IV) on day eight, accompanied by granulocyte growth factor support (G-CSF) on day nine, every 21 days for four cycles in the medical oncology department. She has completed three rounds of chemotherapy and continues to receive treatment with an acceptable level of tolerance.

## Discussion

Uterine leiomyosarcoma is a very rare tumor, representing 1% of all gynecological neoplasms [1]. It is less frequently reported in younger females but is primarily observed in postmenopausal women, with a median age at diagnosis of 56 years and a range of 30 to 73 years for patients with leiomyosarcoma [6]. Only 15% of LMS occur among women less than 40 years of age [7]. 

To our knowledge, our patient is the youngest adult woman documented in the literature. The youngest woman with uterine LMS reported by Begum Monowara was 23 years old [8]. Latika Sahu described another case involving a young patient with primary cervical leiomyosarcoma. The 25-year-old patient underwent initial chemotherapy, surgery, and local vaginal irradiation [9]. Wen-Ying Lee et al. also reported the case of a 28-year-old woman with leiomyosarcoma that developed over pre-existing leiomyomas [10]. 

Even with the standard preoperative evaluation for patients with uterine masses comprising a thorough patient history, physical examination, endometrial sampling, and a variety of imaging techniques, uterine sarcomas are still being unexpectedly discovered during hysterectomy, which presents a significant challenge in differentiating them from benign uterine fibroid tumors [4,6]. 

Uterine leiomyosarcomas (uLMS) are difficult to diagnose preoperatively. In the special report by the American Congress of Obstetrics and Gynecology (ACOG), it is estimated that one in 500 women will have a postoperative diagnosis of stromal sarcoma and leiomyosarcoma [11]. In addition, a review of studies of patients undergoing myomectomy or hysterectomy for presumed benign fibroids reported by the US Food and Drug Administration (FDA) concluded that the risk of occult leiomyosarcoma and sarcoma was one in 458 and one in 352, respectively, in this population [12]. This is the case of our patient, who was diagnosed with leiomyosarcoma postoperatively after a total hysterectomy, which is consistent with the results of the literature. 

The clinical manifestations of leiomyosarcoma are non-specific and do not exhibit substantial distinctions from those of other uterine sarcoma types. Patients frequently arrive with abnormal vaginal bleeding (the most common presenting complaint in 93% of cases), lower abdominal pain or discomfort, a pelvic mass, and a quickly growing fibroid uterus with worsening urinary symptoms [13,14]. Our patient's symptoms included an abdominal mass that rapidly increased in size to 21 centimeters, associated with pain in the lower abdomen. These symptoms were eventually noticed by her family, as the patient was unable to express her illness due to her psychomotor retardation. 

Imaging cannot reliably distinguish malignant from benign causes. Ultrasonography is often the first-line imaging modality for evaluating patients with pelvic disease. Certain ultrasonography characteristics, such as mixed echogenicity, central necrosis, and uneven vasculature on Doppler examination, have been linked to sarcomas. An enlarged uterus, retroverted uterus, and concurrent adnexal processes cannot all be evaluated with this modality, so further imaging techniques were employed [15]. 

The CT appearance of the LMS is not specific. Several CT abnormalities have been reported that are common in patients with LMS, including large masses with central necrosis or liquefaction. The associated metastatic lesion mimics the same characteristics as the primary lesion, particularly in the liver and lungs. In LMS, calcifications are not common [16]. 

When leiomyosarcoma is suspected in females, magnetic resonance imaging (MRI) may be beneficial. Thomassin-Naggara et al. reported that the use of signal intensity (SI) on diffusion-weighted imaging (DWI), SI on T2-weighted MRI, and the ADC value in a multivariate model was accurate in classifying malignant tumors in 92.2% [15,17]. An increased SI on both the high-b-value DWI sequences and the T2-weighted images and an ADC value of less than 1.2 are characteristics of LMS [18]. Research by Lakhman found that the sensitivity and specificity of diagnosing LMS might increase to 95% in the presence of certain features on MR images, such as irregular borders, hemorrhage, areas of decreased SI on T2-weighted images, and central unenhanced areas [19].

The identification of the type of sarcoma before surgery is the most challenging part. An endometrial biopsy is used to diagnose LMS, although it can also be unintentionally found following laparoscopic hysteromyoma surgery of the uterine mass [8,13,14]. 

Under the microscope, the tumor appears as a large, well-circumscribed mass, yellowish-grey in color, with focal hemorrhage and occasionally areas of apparent necrosis. The average diameter of the tumor is 6 cm [20]. LMS are frequently characterized by marked atypia, abundant mitotic activity, and areas of tumor cell necrosis. The presence of two of these three microscopic features is required for the diagnosis of LMS. Hypercellularity and well-defined tumor margins are two additional features typical of leiomyosarcoma [21,22]. Even when tumors with specific features were observed, such as high cellularity, significant hyperchromasia, enormous cells [20], and cellular vacuoles, the diagnosis of LMS was rejected in the absence of mitoses; instead, the tumors were thought to be cellular leiomyoma and bizarre leiomyoma [14]. 

The microscopic characteristics of the LMS are primarily made up of smooth muscle bundles; a single nucleolus or several notable nucleoli give the nuclei a blunted appearance; multinucleated large cells, particularly those of the osteoclastic type, may occasionally be seen [23,24]. The LMS has several morphological subtypes. The most common are myxoid and epithelioid LMS [25,26,27,28]. 

Immunohistochemical analysis of LMS shows positive staining for actin, desmin, caldesmon, ER, PR, and AR. It may also show positive staining for additional epithelial markers such as HMB-45, AE1/AE3, and CAM5.2 and negative staining for CD10 [28,29,30,31]. This was the case in our patient, with positive staining for AML, desmin, and both estrogen and progesterone receptors, as well as negative staining for CD10 and CD117. 

Most LMS is spread quietly by local, regional, or hematogenous routes; locoregional spread results in an abdominal/pelvic mass associated with urinary or gastrointestinal symptoms; and hematogenous spread results in distant metastases [16]. 

According to the European Society for Medical Oncology (ESMO) and National Comprehensive Cancer Network (NCCN) recommendations, the treatment of localized uLMS is en bloc total hysterectomy with bilateral salpingo-oophorectomy followed by clinical observation. Initial surgery should be a wide excision with negative margins (no tumor at the margin, R0). Usually, no pelvic lymph node dissection is performed [32,33]. Aggressive cytoreduction improves the patient's chance of survival. The Dinh TA trial’s results show that patients with LMS had a significant survival difference favoring the group that achieved optimal surgical cytoreduction over the group that was never in surgical remission [21]. The removal of the ovaries and lymph node dissection remain controversial due to the fact that metastases to these organs occur in a small percentage of cases and are frequently associated with intra-abdominal disease [34]. Although less than 12% of patients who underwent lymphadenectomy had lymph node metastases [34,35,36], the decision to perform ovarian conservation may be considered in the early stages (I and II) of the disease for patients under 50 years of age, after a multidisciplinary tumor board review, and with the patient's preference, as there is no significant difference in disease-specific survival, survival, or risk of recurrence with bilateral salpingo-oophorectomy [35]. However, in our patient's case, ovarian conservation was not performed due to the late diagnosis of leiomyosarcoma. The surgeon, with the patient's family's consent, preferred to feel more secure by choosing a bilateral salpingo-oophorectomy in addition to a total hysterectomy to increase the patient's chances of survival. 

It is important to consider the risks of dissemination linked to uterine morcellation [36]. While cell types (epithelioid or spindle), the grade of tumor, the cytologic atypia, or the presence of lymphatic invasion did not prolong survival in multivariate analysis, fewer mitoses per 10 HPF were associated with longer survival [21].

Adjuvant chemotherapy is not a standard treatment and may be proposed as an option for high-risk individual patients after multidisciplinary discussion, taking into account several variables such as high-grade tumors (G2-3), deep tumors > 5 cm, and completely resected leiomyosarcoma (RO) [32,33]. In the adjuvant setting, numerous cytotoxic regimens have been studied, including single-agent ifosfamide or doxorubicin, combined ChT (doxorubicin, ifosfamide, and cisplatin), and gemcitabine/docetaxel. In the Hensley ML trial [37], a total of 25 women with high-grade, uterus-limited uLMS received four cycles of combination adjuvant regimen chemotherapy consisting of docetaxel plus gemcitabine. After a median follow-up of 49 months, 45% of all stage I-IV patients remain progression-free at two years, with a median progression-free survival of 13 months. Analysis of the stage I and II uterine LMS subgroup showed that 59% were progression-free at two and three years, with a median progression-free survival of 39 months and a median overall survival not yet reached. The first sites of relapse were respectively the lung in 3/23 (13%), the pelvis only in 5/23 (22%), and then both pelvis and distant recurrence in five (22%). In our case, the patient is receiving an adjuvant chemotherapy protocol combining intravenous gemcitabine 900 mg/m2 (J1, J8) plus docetaxel 100 mg/m2 (J8) with G-CSF every 21 days for four cycles. During treatment, the courses are well tolerated, with only one episode of grade 2 neutropenia, and the patient has not developed any neurotoxicity to date. She will then be kept under observation.

The effectiveness of adjuvant radiotherapy is controversial and ambiguous; some studies have shown no significant improvement in survival [34,38,39], while others suggest that patients with early-stage malignancies may benefit from adjuvant radiotherapy by having a lower risk of local recurrence [40,41]. As per the Giuntoli RL study, there was no statistically significant difference detected in terms of improved survival between the 31 patients who had adjuvant pelvic radiation therapy and the control group who did not. Gadducci and Larson were able to show that pelvic irradiation may decrease the local recurrence rate without any impact on survival. The local treatment failure rate after surgery was, respectively, 39% and 51%, compared to 33% and 38% after combining surgery with radiotherapy. Adjuvant radiotherapy to the pelvis was given in the immediate postoperative period (starting within eight weeks of surgery) with a total dose of 51 Gy in 28 fractions over five to six weeks, delivering 1.80 Gy per fraction (pelvic radiotherapy, or total abdominal radiotherapy).

LMS is still associated with a poor prognosis. The five-year survival rate for all patients ranges from 13-76%, with over 76% of women with stage I disease confined to the uterus, around 60% of women with stage II disease, and dropping to 10-15% for women with metastatic disease [34,42,43]. 

Recurrence rates vary from 40 to 71%, with a wide range for sites of recurrence [34,43,44]. The recurrence rate for clinical stage I-II LMS was approximately 70%. The most common site of first recurrence is the lung in 40%, followed by the pelvis in 13% [21,43]. Although the time to first relapse varies, it usually occurs within the first two years after diagnosis [42,45]. Patients with metastatic disease typically die within an average of one year after the first relapse has occurred [44].

Surgery for isolated metastatic disease in patients with LMS allowed for a longer disease-free interval and prolonged survival [46]. 

The identification of certain factors, such as the absence of necrosis, peritumoral hyalinization, early-stage disease, and young patient age, as positive prognostic indicators for improved prognosis and extended survival is well-established. Conversely, other factors, including a high mitotic rate, marked atypia, lymph node involvement, large tumor size, lymphovascular space invasion (LVSI), extra-uterine spread, and elderly/post-menopausal status, have been identified as negative prognostic indicators associated with poor outcomes [34,41,47,48,49]. Our patient has several risk factors, including a large tumor size of 21 centimeters and a high mitotic rate of > 10, in addition to moderate atypia, while only young age represents a favorable prognostic factor. Despite these unfavorable factors, the patient remains alive, three months after being diagnosed with uLMS and initiating chemotherapy.

## Conclusions

Uterine leiomyosarcoma is a rare malignancy that rarely affects young adult women. The diagnosis of LMS is typically made using immunohistochemistry. Compared to postmenopausal women, younger patients with LMS tend to have a better prognosis. Surgery is typically the preferred treatment approach, while the role of adjuvant therapy remains a subject of ongoing debate. Despite the standard multimodal treatment strategy, uLMS remains controversial due to the high rates of recurrence and progression. Therefore, close follow-up is strongly recommended to minimize the risk of recurrence.
